# Heart defibrillation: relationship between pacing threshold and defibrillation probability

**DOI:** 10.1186/s12938-019-0715-5

**Published:** 2019-09-13

**Authors:** Priscila C. Antoneli, Jair T. Goulart, Isabella Bonilha, Daniela D. de Carvalho, Pedro X. de Oliveira

**Affiliations:** 1 Department of Biomedical Engineering, School of Electrical and Computer Engineering-FEEC, University of Campinas-UNICAMP, Rua Alexander Fleming 163, Cidade Universitária Zeferino Vaz, Campinas, SP CEP 13083-881 Brazil; 2 Department of Physiological Sciences, Institute of Biology, University of Brasilia-UnB, Campus Universitário Darcy Ribeiro-Asa Norte, Brasília, DF CEP 70910-900 Brazil; 3 Laboratory of Myocardial Ischemia/Reperfusion, Faculty of Medical Science, University of Campinas-UNICAMP, Rua Cinco de Junho, 350, Bloco 1, Cidade Universitária Zeferino Vaz, Campinas, SP CEP 13083-877 Brazil; 4 Center for Biomedical Engineering, University of Campinas-UNICAMP, Rua Alexander Fleming 163, Cidade Universitária Zeferino Vaz, Campinas, SP CEP 13083-881 Brazil

**Keywords:** Ventricular fibrillation, Defibrillation, Electric stimulation, Isolated rat heart preparation

## Abstract

**Background:**

Considering the clinical importance of the ventricular fibrillation and that the most used therapy to reverse it has a critical side effect on the cardiac tissue, it is desirable to optimize defibrillation parameters to increase its efficiency. In this study, we investigated the influence of stimuli duration on the relationship between pacing threshold and defibrillation probability.

**Results:**

We found out that 0.5-ms-long pulses had a lower ratio of defibrillation probability to the pacing threshold, although the higher the pulse duration the lower is the electric field intensity required to defibrillate the hearts.

**Conclusion:**

The appropriate choice of defibrillatory shock parameters is able to increase the efficiency of the defibrillation improving the survival chances after the occurrence of a severe arrhythmia. The relationship between pulse duration and the probability of reversal of fibrillation shows that this parameter cannot be underestimated in defibrillator design since different pulse durations have different levels of safety.

## Background

Life-threatening arrhythmias (LTA) such as ventricular fibrillation (VF) are very serious conditions that may lead to death in few minutes. VF is characterized by chaotic and asynchronous cardiomyocyte electrical activity which leads to ineffective heart pumping [1]. It has a prevalence of approximately ~ 25–50% of people with out-of-hospital cardiac arrest (OHCA) [2–4]. LTA are one of the major causes of death around the world. Annually, 35 per 100,000 people experience OHCA globally, including adults and children, and this number increases to 62 per 100,000 people when only adults are taken in account [5].

Once LTA are diagnosed, a high-intensity electric field (HEF) must be applied in the patient as soon as a defibrillator is available in a procedure called defibrillation [6]. For effective defibrillation, a critical mass (75–90%) of ventricular cardiomyocytes has to be excited at the same time [7]. However, the excitation of this large number of cardiomyocytes requires the application of HEF which reaches around 100 V/cm or higher in some regions of the myocardium [8]. A HEF of this magnitude may lead to acute myocardial injury by electroporation [9], depression of contractile function [10] and blockage of electrical conduction by necrosis [11]. Furthermore, our research team has already demonstrated that HEF of such intensity is able to kill cardiomyocytes [12–14]. Nevertheless, even a non-lethal HEF can make the cell unexcitable, generating a substrate to arrhythmia re-induction [15]. These side effects might be related to the low survival rates reported after OHCA, which have been stable at 7–8% for the last 30 years despite the improvements in treatment and the increased availability of automated external defibrillators (AEDs) in public places [4, 16]. In this context, several studies have been carried out with the aim of improving defibrillation procedure to increase its success rate whilst reducing its side effects.

Our aim was to show the efficacy of a simple and feasible method able to improve the defibrillatory procedure, through the study of the strength–duration (SxD) curves. SxD curves have been exhaustively studied for the heart, but for the first time we present a paired study with heart pacing SxD curves and defibrillation SxD curves for the same hearts; from these data, we also propose a relationship between heart pacing electric field (E) and defibrillation HEF as a possible indicator of heart damage risk.

Also, a previous study of our research team has shown that the ratio of lethal HEF to excitation threshold for isolated rat cardiomyocytes changes with stimuli duration and is maximal for 0.5-ms stimuli [12], which indicates that this duration would probably be safer for defibrillation. In this study, we confirmed the relationship between defibrillation safety and pulse duration through SxD curves. We correlated the required HEF intensity for defibrillation with the shock intensity required for heart pacing to verify whether the existence of a previously observed optimum duration for cardiomyocyte stimulation would be translated to a higher efficiency in rat heart defibrillation.

## Results

Adult male Wistar rats were euthanized under deep anesthesia and the hearts were removed and cannulated in less than 30 s, avoiding physiological function loss due to prolonged ischemia [17, 18]. Hearts weighed on average 2.46 ± 0.07 g.

### Pacing threshold

**Fig. 1:**
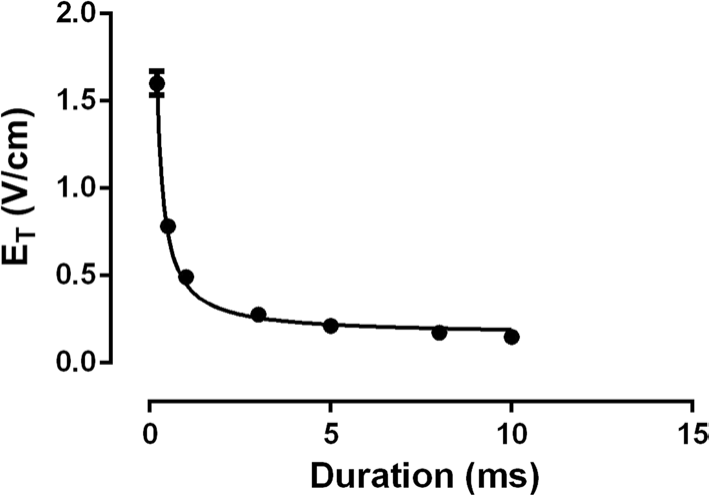
Pacing strength–duration curve. *R*^2^ = 0.9609 (Weiss–Lapicque equation). Circles indicate the mean pacing threshold (*E*_T_) and the vertical lines indicate standard error of the mean (SEM). Curve was fitted by Eq. (1) (rheobase = 0.16 ± 0.01 V/cm, chronaxie = 1.820 ± 0.20 ms), *n* = 10

Pacing strength–duration (SxD) curve (Fig. 1) shows the correlation between the stimulatory pulse duration and the mean pacing threshold (*E*_T_). The SxD curve was well adjusted by the Weiss–Lapicque equation (Eq. 1, *R*^2^ = 0.96), where *Y* is the HEF intensity corresponding to a pulse duration *d*, *E*_rh_ is the rheobase value (field modulus when *d* is infinity) and *c*_r_ is the chronaxie (pulse duration corresponding to twice the rheobase). Chronaxie and rheobase values were 1.820 ± 0.20 ms and 0.16 ± 0.01 V/cm, respectively,

1 $$Y = E_{{{\text{rh}} }} \left( {1 + \frac{{c_{\text{r}} }}{d}} \right) .$$

### Defibrillation probability curves

**Fig. 2:**
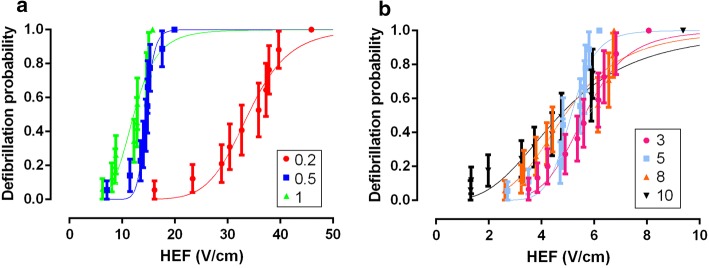
Defibrillation probability as a function of the applied electric fields (V/cm). Vertical lines indicate the standard error of the mean (SEM). **a** Curves of the durations 0.2-; 0.5- and 1-ms. **b** Curves of durations 3-, 5-, 8- and 10-ms. Curves were fitted by Eq. (3), *n* = 10, each heart was defibrillated seven times (one time for each pulse duration)

**Fig. 3:**
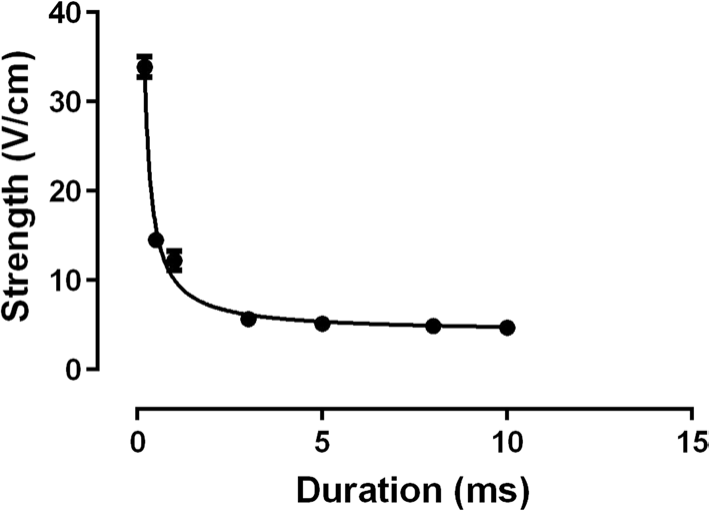
Defibrillation probability strength–duration curve. *R*^2^ = 0.9885 (Weiss–Lapicque equation). Points indicate the electric field associated with 50% of defibrillation success (HEF_50_) and the vertical lines indicate the confidence interval for 95%. Curve was fitted by Eq. (1) (rheobase = 4.17 ± 0.561 V/cm, chronaxie = 1.41 ± 0.235 ms), *n* = 10, each heart was defibrillated seven times (one time for each pulse duration)

Figure 2 shows the defibrillation probability curves as a function of the applied HEF in V/cm for all tested durations. These curves are significantly different (*p* < 0.0001). When compared in pairs, the 0.2-ms curve was different from all the others, whilst 0.5-ms and 1-ms curves were similar but different from all other curves. The average values of HEF corresponding to 50% of defibrillation probability (HEF_50_) values in V/cm were obtained from the nonlinear fit of the survival test results (Fig. 2) and were significantly different from each other (*p* < 0.0001). The HEF_50_ of curves with duration of 0.2-, 0.5-, 1- and 3- ms was different from all the others. On the other hand, 5-, 8- and 10-ms curves presented very close HEF_50_ values with no significant differences between them. Then the increase in the defibrillatory pulse duration beyond 5-ms did not promote intensity reduction of the shocks required for defibrillation. The adjustment of these HEF_50_ values by the Weiss–Lapicque equation generated a defibrillation SxD curve (Fig. 3) with rheobase and chronaxie of the 4.17 ± 0.561 V/cm and 1.41 ± 0.235 ms, respectively.

### Ratio of defibrillation probability to pacing threshold

**Fig. 4:**
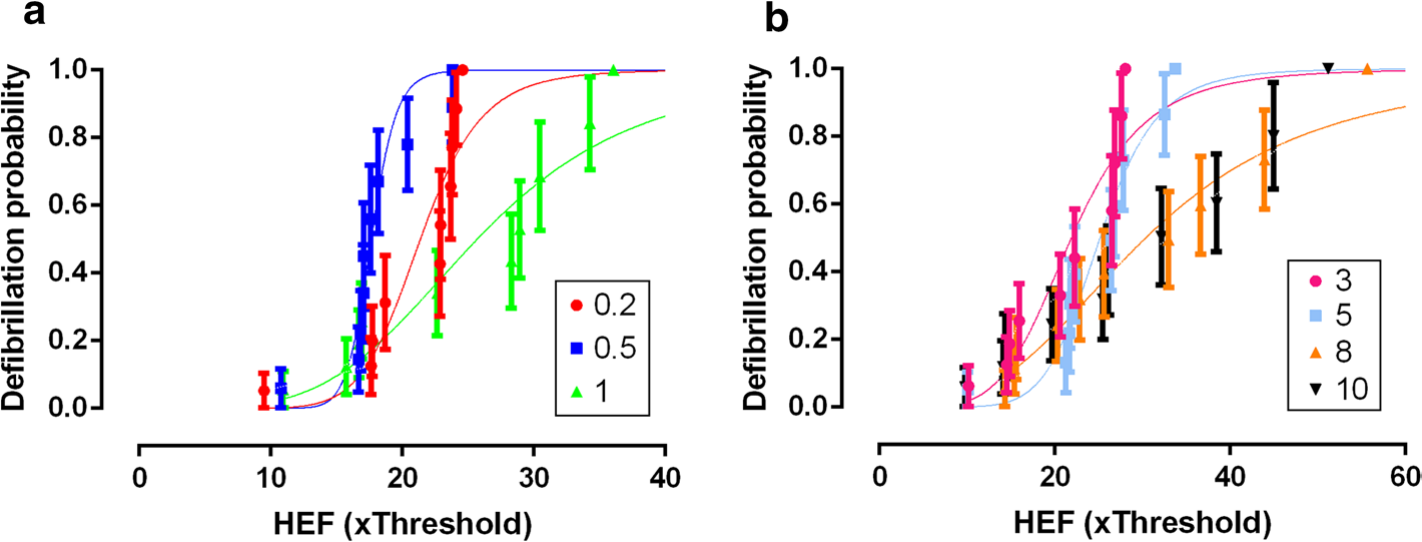
Curves obtained from the sigmoidal adjustment of the normalized defibrillation probability result as a function of the electric field (×Threshold). Vertical lines indicate standard error of the mean (SEM). **a** Curves of the durations 0.2-, 0.5- and 1-ms. **b** Curves of durations 3-, 5-, 8- and 10-ms. Curves were fitted by Eq. (3), *n* = 10

**Fig. 5:**
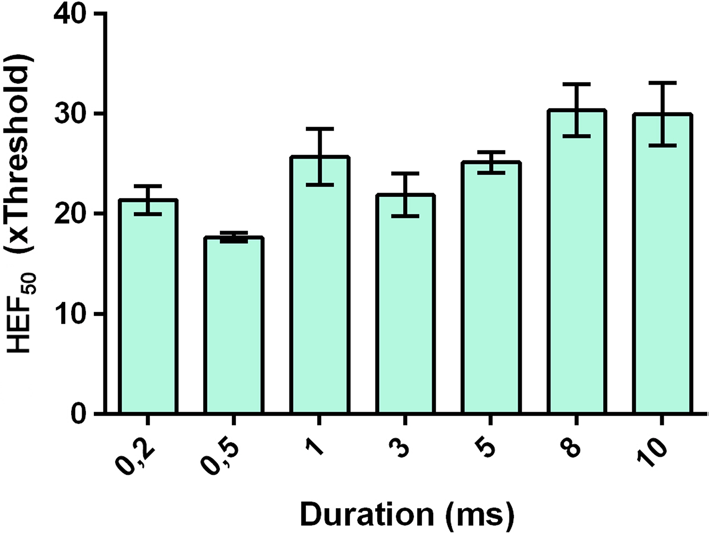
Pacing threshold and defibrillation probability relationship. The bars indicate the mean value of HEF_50_ normalized by the pacing threshold and the vertical lines indicate the confidence interval for 95%, *n* = 10

The HEF_50_ obtained from the probability curves (Fig. 4) for each pulse duration is shown in Fig. 5. This graphical representation puts emphasis on how greater than the pacing threshold a shock should be to succeed in defibrillation. In general, the HEF_50_ was over 20 times the threshold (×Threshold), the only exception was the 0.5-ms pulses, which HEF_50_ was 17.65 ×Threshold. Normalized HEF_50_ were different (*p* < 0.001) and when compared in pairs, the 0.2-ms pulse was not different from 0.5-ms and 3-ms pulses, but it was different from all the others. 1-ms pulse was different from the 3-ms, and 3-ms was different from the 8-ms pulse. The HEF_50_ values of the sigmoid adjustment were also significantly different (*p* < 0.001). When compared in pairs, the HEF_50_ of the 0.5-ms curve (the only one below 20 ×Threshold) is different from all others. There was no difference in the comparison between the HEF_50_ of the 0.2-ms and 3-ms curves, between the 1-ms and 5-ms curves, and the 8-ms and 10-ms curves.

**Fig. 6:**
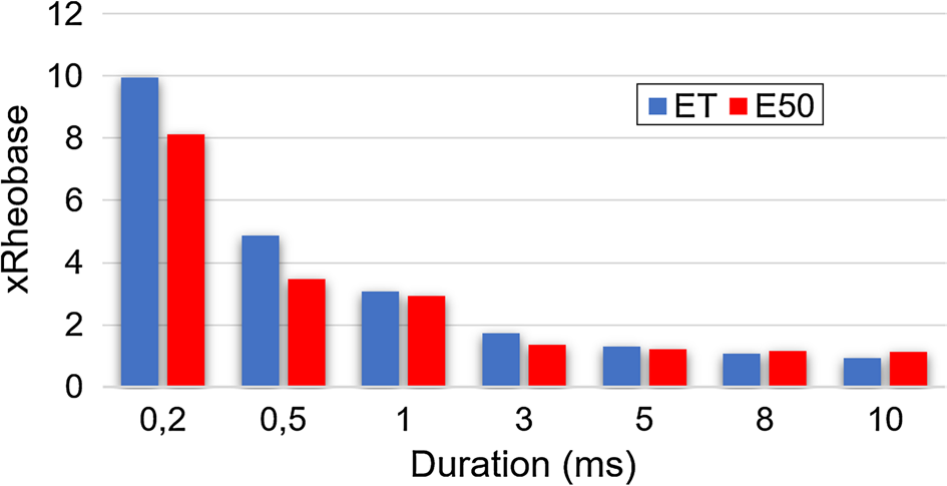
Ratio of *E*_T_ and HEF_50_ to rheobase. The value of HEF_50_ to 0.5-ms varied less than *E*_T_ regarding to rheobase, *n* = 10

To better understand this last result, we normalized the SxD curves (SxD for pacing—Fig. 1, and SxD for defibrillation—Fig. 3) to their respective rheobase (Fig. 6). For durations of 10-, 8-, 5-, 3- and 1-ms, the values of rheobase normalized *E*_T_ and HEF_50_ are similar. For durations below 3-ms, we observed that not only both *E*_T_ and HEF_50_ values increase, but also the difference between them. For 1- to 0.5-ms, the HEF_50_ increase was smaller (18%) than the *E*_T_ increase (58%). For 0.5- to 0.2-ms, both HEF_50_ and *E*_T_ values increased similarly, about 104% and 134%, respectively; therefore, the HEF_50_/*E*_T_ ratio is lower for 0.5-ms since the *E*_T_ variation is greater than the HEF_50_ for this duration, whereas it is not so pronounced for the others.

## Discussion

The present study shows that, within certain limits, the longer the pulse duration is, the lower is the threshold intensity for pacing and defibrillation, as expected for the stimulation of excitable tissues [19, 20]. Herein, we show for the first time, to the best of our knowledge, the stimulation and defibrillation SxD curves for the same hearts, considering an applied E homogeneously, which generated values more reliable and preparation independent.

We observed the same behavior for SxD curves for pacing and for defibrillation. Pacing requires a small pulse strength to be successful, because when a small number of cells are excited, the action potential propagation occurs throughout the heart [21]. Thus, only a small number of cells need to be submitted to a supra-threshold *E*. In contrast, for defibrillation, a simultaneous excitation of a large portion of the myocardium (75–90%) [7] is required to make the cells non-excitable for a period and to terminate the fibrillatory mechanisms.

During the *E* application, non-uniform potential gradient formation happens because the cardiac tissue is anisotropic, composed by muscle fibers oriented in multiple directions with layers of connective tissue [22, 23]. Also, the heart region subjected to a higher potential gradient is closer to the electrodes; consequently, this region is easily stimulated, while other regions might not be stimulated depending on the applied *E* strength. However, when a critical mass of cardiomyocytes must be depolarized at the same time, as in the case of defibrillation, *E* has to be increased to stimulate cells which are not close to the electrodes; as a result, defibrillatory *E* is much larger than *E*_T_ [21, 24]. In addition, pacing occurs during diastole, when most ventricular myocytes are relaxed in a vulnerable period. However, during defibrillation, the myocytes are not synchronized, each group of cells may be in a different action potential phase, requiring even higher amplitudes to excite cells during their relative refractory period and then terminate the fibrillation wave fronts [22, 24]. Hence, during defibrillation, the closest regions to the electrodes are exposed to a much higher *E* than the *E*_T_. The transmembrane potential variation (Δ*V*_m_) of each myocyte is proportional to the applied *E* module [25], then the maximal Δ*V*_m_ is observed in the near-electrode myocytes; moreover, during threshold pacing, we may assume that the maximal Δ*V*_m_ in the myocytes of this region is the stimulation threshold (Δ*V*_mT_); as a result, it is constant and does not change according to stimuli duration [26]. During defibrillation, the Δ*V*_m_ can be expressed by HEF_defibrillatory_/*E*_T_ multiplied by the Δ*V*_mT_, where HEF_defibrillatory_ is the high-intensity electric field necessary to successful defibrillation, which means that the HEF_defibrillatory_/*E*_T_ could be taken as an indirect index of the induced Δ*V*_m_ in the cardiomyocytes during defibrillation. This information is very important because it allows to infer which duration induced a lower Δ*V*_m_, since a high Δ*V*_m_ may lead to electroporation and consequent cell death [27, 28]. We observed a lower HEF_defibrillatory_/*E*_T_ ratio (17.65) when we defibrillated with 0.5-ms pulses. Thus, using this pulse duration, the induced Δ*V*_m_ in the cardiomyocytes was probably lower and, consequently, it may be safer to be used in defibrillation procedures.

Defibrillatory pulses with duration of 0.5-ms are probably better for defibrillating rat hearts since not only the HEF_50_ in ×Threshold is smaller, but cells are also less susceptible to injury for this duration [12]. Although the defibrillation success × pulse duration depends on the animal study [24, 29], the use of a short pulse duration might improve defibrillation procedures in human hearts, as Semenov et al. [30] also argued, since the commercial defibrillators use pulses with 5- or 10-ms duration, i.e., near the rheobase [21, 22]. Despite the difference in heart size between rodents and human, a factor that can influence the cardiac arrest mechanisms [31], models using rodent hearts have several advantages as presented by Patten et al. [32]. These models, such as the one used in this study, produce results that cannot be directly related to the clinical context, but that generate important results, especially on the understanding, diagnosis and treatment of conditions such as VF because of the unavailability of studies on human subjects for ethical reasons. However, due to the limitations of the models, for results of basic science to be translated into clinical practice, studies in larger mammals, whose heart size is more similar to that of humans, are needed.

A possible limitation of this work was the time between heart removal and cannulation finalization (30 s). However, it was not sufficient to cause ischemia impairment in previous studies [17, 18]; additionally, contractile and chronotropic impairment may be present due to prolonged experiment time and cumulative effect of consecutive shocks.

Despite the fact that the rat hearts were placed in a Langendorff-adapted preparation for a maximum time of 3 h and that this type of preparation leads to contractile and chronotropic function deterioration of the heart ranging from 5 to 10% per hour [17], we believe that the randomized choice of the pulse duration sequence could minimize changes in the outcomes that were implied by this deterioration. However, we did not note any significant change in the heart function during the experiments involving all hearts included in this work.

We hope that this work can bring important clinical implications in the future, leading to an optimization of commercial defibrillators only by changing the pulse duration. A simple reduction of the shock duration, even on a small scale, may possibly lead to a significant increase in the effectiveness of defibrillatory procedures.

## Conclusions

Considering our results, it is possible to conclude that defibrillated rat hearts by 0.5-ms pulses are less likely to suffer from injuries since the relationship between defibrillation probability and pacing threshold was lower for this duration, indicating that the impairment is smaller because the induced potential is lower in this case.

This outcome, along with a greater stimulatory safety factor for the duration of 0.5-ms [12], supports the hypothesis that a defibrillatory shock with this duration would be better for reversing VF in rats. Still, further studies should be performed to identify possible mechanisms underlying this finding.

## Materials and methods

The protocols for animal care and use were approved by the Institutional Committee for Ethics in Animal Research (IB/UNICAMP, No. 4355-1). All the animals received care in accordance with relevant guidelines and regulations.

### Isolated heart preparation

**Fig. 7:**
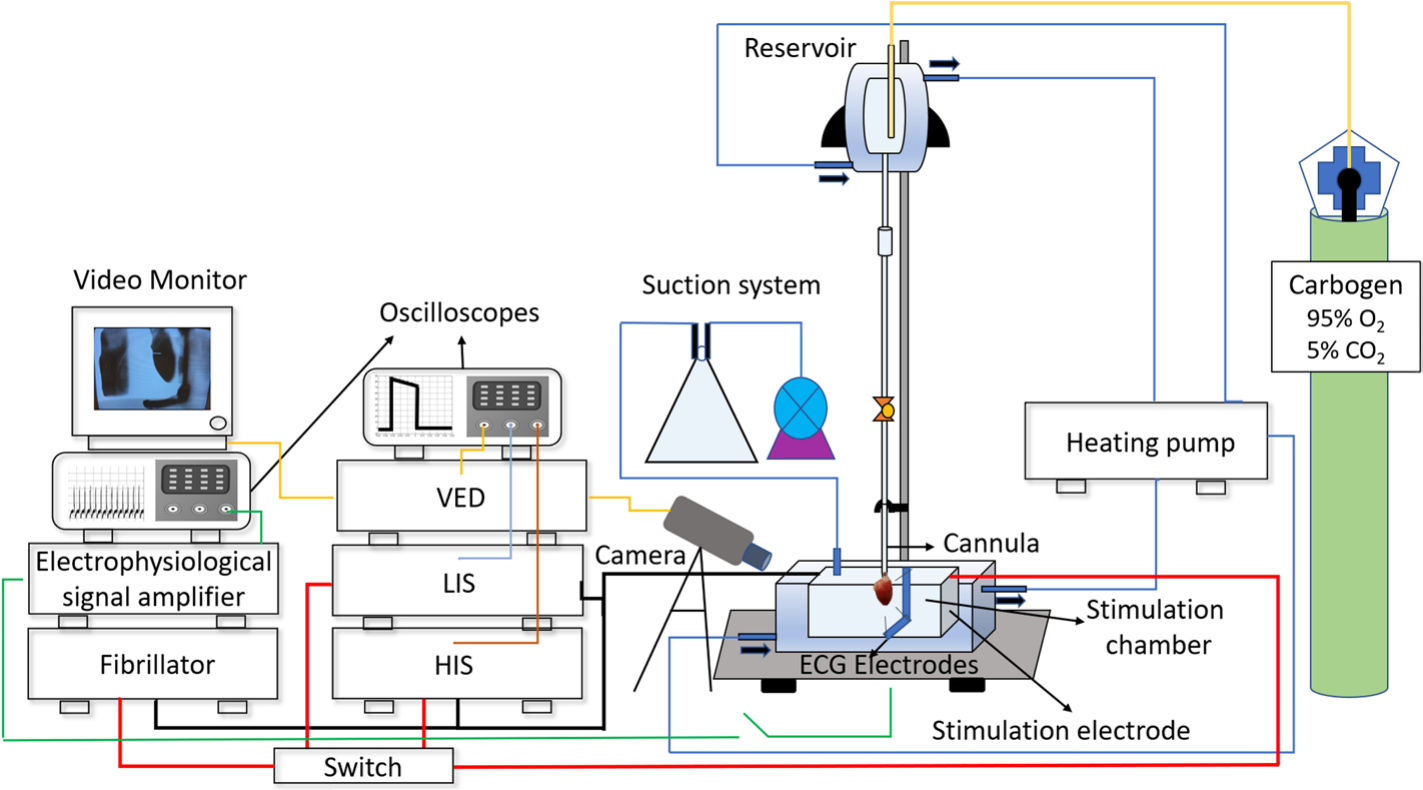
Experimental setup diagram. VED is the video edge detector, LIS is the low-intensity stimulator and the HIS is the high-intensity stimulator

Ten male Wistar rats with age ranging from 5 to 6 months and average weight of 535.3 g ± 6.4 g were used. The animals received intraperitoneal injection of sodium heparin (3000 IU/kg), and they were anesthetized with an anesthetic button of lidocaine (5 mg/kg) and with an intraperitoneal injection of thiopental sodium (80 mg/kg). Following the chest opening, the heart was quickly and carefully excised. The aorta was cannulated in a Langendorff-adapted preparation (Fig. 7), where the heart was retrogradely perfused with Krebs–Henseleit solution composed of salts with the following concentrations (mM): NaCl 115, NaHCO_3_ 25, KCl 4.6, MgSO_4_ 1.2 KH_2_PO_4_ 1.2, glucose 11.0 and CaCl_2_ 1.4, constantly carbonated with carbogen mixture (5% CO_2_ and 95% O_2_) to maintain the pH between 7.35 and 7.45, and heated to 37 °C ± 0.5 °C.

### Experimental protocol

After cannulation, the hearts were housed in the internal reservoir of the stimulation chamber (Fig. 7), heated (37 °C) by hot water circulation in the external reservoir provided by a pump (developed and manufactured by the Center for Biomedical Engineering, Campinas, Brazil). The heart was positioned in the middle of the chamber, 4 cm distant from the parallel plates located on the sides of the stimulation chamber, i.e., the stimulation electrodes, what allowed a uniform distribution of HEF within the chamber [33], which can be calculated according to the following equation [34]:

2 $$E = \frac{I}{{\sigma \cdot {\text{h}} \cdot {\text{w }}}},$$

where *E* is the electric field module, *I* is the current flowing through the chamber, *σ* is the physiological solution conductivity, *h* is the submerged height of the electrodes and *w* is the electrode width. In our setup, the conductive bath had 3.9 cm height (*h*) per 10.0 cm width (*w*), constituting a total volume of 152 ml and calculated resistance of 45.06 Ω. The Krebs–Henseleit solution *σ* was assumed to be 0.014 S/cm [34].

After 10 min for heart rate stabilization, the cardiac electrophysiological signal (ECG) was captured by Ag/AgCl electrodes (ECG electrodes), amplified (gain = 2000) and filtered (high-pass filter: *f*_ch_ = 3 Hz; low-pass filter: *f*_cl_ = 100 Hz) by a electrophysiological signal amplifier (developed and manufactured by the Center of Biomedical Engineering, Campinas, Brazil). The ECG trace was visualized in an oscilloscope (manufactured by Tektronix Inc. Beaverton, OR, USA, model TDS 2014C, 100 MHz bandwidth) (Fig. 7). The spontaneous heart rate was determined by measuring the interval between five ECG R-waves.

Stimulation electrodes were connected to a low-intensity electrical stimulator (LIS, developed and manufactured by Center for Biomedical Engineering, Campinas, Brazil). The pacing threshold was determined for seven pulse durations (0.2-, 0.5-, 1-, 3-, 5-, 8- and 10-ms, total wave duration). The pulse duration sequence was randomly chosen for each heart and the stimulus frequency was set to 20% above the measured spontaneous heart rate. The stimulus amplitude was increased until the heart rate was equalized with the stimulation rate; the heart rate was inferred through the use of a video signal edge detector (VED, developed and manufactured by the Center for Biomedical Engineering, Campinas, Brazil). VED was coupled to a video camera (Ikegami Tsushinki Co., LTD, Japan—ICD-31 mod.) and to a video monitor (Kodo Electronics Co, LTD, Seoul, Korea—mod. KBM1200S, Fig. 7). The voltage output of the VED was proportional to the displacement of the heart border. When the electrical output signal of the VED synchronized with the stimulatory pulses, we considered that the heart was being paced. The minimum electric field (*E*) that kept the synchronism was considered the *E*_T_. This protocol was repeated for each stimuli duration.

The fibrillator (developed and manufactured by the Center for Biomedical Engineering Center, Campinas, Brazil) was coupled to the stimulation electrodes and the VF was induced by delivering a sine wave signal, with 60 Hz, amplitude from 1 to 3 V/cm and duration from 0.5 to 2 s [10, 35]. Duration and stimuli amplitude were adjusted to induce VF which was detected by monitoring the ECG record. When VF was maintained for at least 2 min, it was considered sustained and the fibrillator was disconnected; otherwise, a new amplitude and duration combination was set and VF was re-induced.

**Fig. 8:**
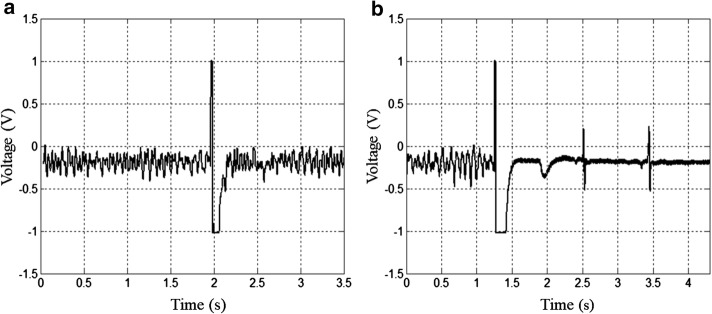
Defibrillation evaluation. Electrocardiogram analysis after defibrillatory pulse application. **a** Defibrillation failure (the signal between 2 and 2.2 s was caused by the defibrillatory pulse application) and **b** defibrillation success (the signal between 1.3 and 1.45 s was caused by the defibrillatory pulse application)

Once sustained VF was confirmed, the defibrillation protocol was started. A high-intensity electrical stimulator (HIS, developed and manufactured by the Center for Biomedical Engineering, Campinas, Brazil) was coupled with the stimulation electrodes and a monopolar electrical stimuli was applied with the truncated exponential waveform (decay less than 10%, with variable voltage from 1 to 1000 V and duration from 0.2- to 10-ms). The pulse duration was randomly chosen and the amplitude was initially set to five times the *E*_T_ for the chosen duration of the same heart. This procedure was repeated for stimuli amplitudes between 10 and 35 times *E*_T_, or until defibrillation was confirmed, i.e., return of a clear QRS complex on the ECG record combined with heart contraction (Fig. 8, square A shows a case of defibrillation failure, and square B shows a case of success). For each heart, the procedures for fibrillation and defibrillation were performed once for each pulse duration, with intervals of 5 min to stabilize heart rate. The sequence of pulses with different durations was randomly chosen for each heart.

### Statistical analysis

Defibrillation probability curves were based on the relationship between defibrillation probability and applied HEF in V/cm and ×Threshold (HEF values applied normalized to *E*_T_) by survival analysis [36]. The curves obtained were compared by the Mantel–Cox test and adjusted by the following equation:

3 $$L\left( {\text{HEF}} \right) = \frac{1}{{1 + \left( {\frac{{{\text{HEF}}_{50} }}{\text{HEF}}} \right)^{h} }},$$

where *L* is defibrillation probability, HEF_50_ is the average value of HEF corresponding to 50% of defibrillation probability and h is the Hill coefficient [14]. HEF_50_ values in V/cm and ×Threshold for each duration were compared by *F* test.

Two SxD curves were obtained: pacing SxD curve was made with *E*_T_ values and the defibrillation probability SxD curve was plotted with the average values of HEF_50_, in V/cm obtained from the survival analysis. Both SxD curves were adjusted by Weiss–Lapicque equation (Eq. 1).

The ratio of defibrillation probability to pacing threshold was plotted with the average values of HEF_50_, in ×Threshold obtained from the survival analysis.

Statistical significance index *α* adopted for all tests was 0.05. All analyses and tests were made with the software Prism 5.03 (GraphPad Software, San Diego, US).

## Data Availability

The datasets used and/or analyzed during the current study are available from the corresponding author on reasonable request.

## Abbreviations

Δ*V*_m_: transmembrane potential variation
Δ*V*_mT_: transmembrane potential variation threshold
AEDs: automated external defibrillators
*c*_r_: chronaxie
*E*: electric field
*E*_rh_: rheobase value
*E*_T_: pacing threshold
*f*_ch_: high-pass filter
*f*_cl_: low-pass filter
*h*: height
HEF: high-intensity electric field
HEF_50_: the average value of HEF corresponding to 50% of defibrillation probability
HEF_defibrillatory_: high-intensity electric field necessary to successful defibrillation
HIS: high-intensity electrical stimulator
LIS: low-intensity electrical stimulator
LTA: life-threatening arrhythmias
OHCA: out-of-hospital cardiac arrest
SEM: standard error of the mean
SxD: strength–duration
VED: video signal edge detector
VF: ventricular fibrillation
*w*: width
